# Oxidative stress resulting from the removal of endogenous catalase induces obesity by promoting hyperplasia and hypertrophy of white adipocytes

**DOI:** 10.1016/j.redox.2020.101749

**Published:** 2020-10-10

**Authors:** Su-Kyung Shin, Hyun-Woo Cho, Seung-Eun Song, Seung-Soon Im, Jae-Hoon Bae, Dae-Kyu Song

**Affiliations:** Department of Physiology & Obesity-mediated Disease Research Center, Keimyung University School of Medicine, Daegu, 42601, South Korea

**Keywords:** Catalase, Lipogenesis, White adipocyte, NOX4, AMPK, Obesity, Acox1, acyl-CoA oxidase 1, AICAR, 5-aminoimidazole-4-carboxamide ribonucleotide, AMP, adenosine monophosphate, AMPK, AMP-activated protein kinase, ATP5A, ATP synthase alpha-subunit gene, C/EBP, CCAAT/enhancer-binding protein, CKO, catalase-knockout, CPT, carnitine palmitoyltransferase, Cs, citrate synthase, DMEM, Dulbecco's Modified Eagle Medium, ETC, electron transport chain, FAS, fatty acid synthase, FBS, fetal bovine serum, FER, food efficiency ratio, Fgf21, fibroblast growth factor 21, GAPDH, glyceraldehyde 3-phosphate dehydrogenase, GATA2, GATA-binding protein 2, G6PD, glucose-6-phosphate dehydrogenase, H&E, hematoxylin and eosin, HFD, high-fat diet, HIF1α, hypoxia-inducible factor 1α, IDH, isocitrate dehydrogenase, Lcad, long-chain acyl-CoA dehydrogenases, Mcad, medium-chain acyl-CoA dehydrogenase, MDH, malate dehydrogenase, MEF, mouse embryonic fibroblast, MTCO1, mitochondrially encoded cytochrome C oxidase I, NAC, *N*-acetyl cysteine, ND, normal chow diet, NDUFB8, NADH dehydrogenase [ubiquinone] 1 beta subcomplex subunit 8, mitochondrial, NOX, NADPH oxidase, NRF1, nuclear respiratory factor 1, ORO, Oil Red O, OXPHOS, total oxidative phosphorylation system, PASMC, pulmonary artery smooth muscle cells, PBS, phosphate-buffered saline, PGC1α, PPARγ coactivator 1-α, PPARγ, peroxisome proliferator-activated receptor γ, PPP, pentose phosphate pathway, Pref-1, preadipocyte factor 1, qPCR, quantitative polymerase chain reaction, ROS, reactive oxygen species, SDH, succinate dehydrogenase, SDHB, succinate dehydrogenase complex iron sulfur subunit B, SEM, standard error of the mean, siCAT, catalase-siRNA, siRNA, small interfering RNA, SREBP, sterol regulatory element-binding protein, TCA, tricarboxylic acid, TFAM, mitochondrial transcription factor A, TG, triglycerides, UQCRC, ubiquinol-cytochrome C reductase core protein 2, WAT, white adipose tissue, WT, wild-type

## Abstract

Obesity is regarded as an abnormal expansion and excessive accumulation of fat mass in white adipose tissue. The involvement of oxidative stress in the development of obesity is still unclear. Although mainly present in peroxisomes, catalase scavenges intracellular H_2_O_2_ at toxic levels. Therefore, we used catalase-knockout (CKO) mice to elucidate the involvement of excessive H_2_O_2_ in the development of obesity. CKO mice with C57BL/6J background gained more weight with higher body fat mass with age than age-matched wild-type (WT) mice fed with either chow or high-fat diets. This phenomenon was attenuated by concomitant treatment with the antioxidants, melatonin or *N*-acetyl cysteine. Moreover, CKO mouse embryonic fibroblasts (MEFs) appeared to differentiate to adipocytes more easily than WT MEFs, showing increased H_2_O_2_ concentrations. Using 3T3-L1-derived adipocytes transfected with catalase-small interfering RNA, we confirmed that a more prominent lipogenesis occurred in catalase-deficient cells than in WT cells. Catalase-deficient adipocytes presented increased nicotinamide adenine dinucleotide phosphate oxidase 4 (NOX4) expression but decreased adenosine monophosphate-activated protein kinase (AMPK) expression. Treatment with a NOX4 inhibitor or AMPK activator rescued the propensity for obesity of CKO mice. These findings suggest that excessive H_2_O_2_ and related oxidative stress increase body fat mass via both adipogenesis and lipogenesis. Manipulating NOX4 and AMPK in white adipocytes may be a therapeutic tool against obesity augmented by oxidative stress.

## Highlights

• Catalase deficiency facilitates both adipogenesis and adipocyte hypertrophy.
• Catalase deficiency activates NOX4, resulting in further oxidative stress increase.
• Catalase KO-induced HIF1α activation and pentose phosphate pathway further activate NOX4.
• Catalase deficiency inhibits AMPKα, which is attributed to mitochondrial dysfunction.
• Antioxidants, NOX4 inhibitors, and AMPKα activators prevent obesity.

## Graphical abstract

**Figure:**
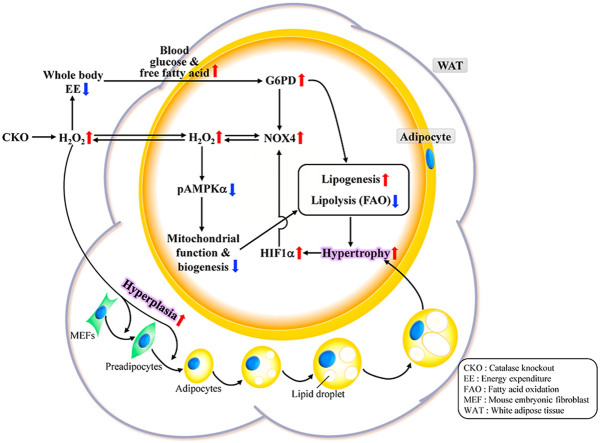

## 1 Introduction

Adipocytes are the main components of adipose tissue and associated with numerous physiological and pathological metabolic events. Adipocytes can be used as a measure of obesity based on their size and number. Hence, controlling the differentiation of embryonic stem cells into the preadipocyte lineage and of preadipocytes to adipocytes (hyperplasia, adipogenesis) and the hypertrophy (lipogenesis) of mature adipocytes would be an effective strategy to manipulate body adiposity, and thus obesity. Adipocyte formation is a process enclosing six specific orchestrated steps [1] but is broadly divided into two phases. The first phase is the differentiation of mesenchymal precursors, which can differentiate into multiple lineages, to preadipocytes, while the second phase consists of the differentiation of preadipocytes into mature adipocytes. The first phase is mainly affected by the Wnt/β-catenin signaling pathway, which facilitates the differentiation of mesenchymal precursors into osteoblasts, while inhibiting that into preadipocytes [[1], [2], [3]]. The second phase involves a cascade of transcription factors, among which peroxisome proliferator-activated receptor γ (PPARγ) and CCAAT/enhancer-binding proteins (C/EBPs) are considered important marker of adipocytes at early-stage differentiation [1,4,5].

Studies on the effect of reactive oxygen species (ROS) on mesenchymal stromal cells and their precursors have reported that ROS primarily affect their survival [6]. Remarkably, a report demonstrated that treatment with exogenous H_2_O_2_ suppresses Wnt signaling, resulting in reduced osteoblastogenesis but promoting adipogenesis [7]. Furthermore, treatment with H_2_O_2_ facilitates the proliferation and differentiation of 3T3-L1 cells [8,9]. Recently, various studies using *in vitro* models have also proposed and proven that H_2_O_2_ promotes lipogenesis in adipocytes via mimicking insulin [[9], [10], [11]].

Catalase is a well-known cellular antioxidant enzyme, mainly expressed in peroxisomes, that eliminates excessive H_2_O_2_. Acatalasemia refers to a genetic deficiency in erythrocyte catalase activity that increases H_2_O_2_ concentration in tissues, as glutathione peroxidase activity does not compensate for the lack of catalase [[12], [13], [14]]. Although this syndrome was initially thought to be asymptomatic, it has been recognized as a risk factor for age-related diseases in humans, such as diabetes, hypertension, Alzheimer's disease, neoplasms, and atherosclerosis [15,16]. Particularly, Góth et al. reported a high prevalence of diabetes in Hungarians with catalase deficiency [14]. Pancreatic β-cells are poor in catalase but rich in mitochondria, which are susceptible to damage by oxidizing species emanated from the blood [17,18]. In C57BL/6J mice, catalase deficiency also promotes diabetes and has been reported to significantly increase body weight [19,20]. In humans, it is unclear whether catalase deficiency is associated with obesity; however, this seems to be true, at least partially, as demonstrated by the cautious lifestyle lead by these patients to prevent complications from acatalasemia, including diabetes. Many articles demonstrated that obesity or excessive adipose tissue mass results in an excessive production of ROS, which triggers systemic metabolic disturbances, such as insulin resistance and cardiovascular complications [21,22]. Nevertheless, only few studies have elucidated the ROS-mediated mechanisms underlying the development of obesity. In this study, we investigated the involvement of excessive H_2_O_2_ in the development of obesity, taking advantage of catalase-knockout (CKO) mice, and the underlying molecular mechanisms.

## 2 Materials and methods

### 2.1 Animals and treatment

Male C57BL/6J mice (wild-type, WT) were purchased from Jung Ang Experimental Animals (Seoul, Korea). Male CKO mice were derived from a catalase-null line generated by Ho et al. [23]. All mice used in experiments were acclimated for a week and housed under controlled 12 h dark-light cycles and constant temperature (25 °C). During the breeding period, body weight and food intake were periodically measured.

1 Four-week-old male C57BL/6J (n = 10) and CKO (n = 10) mice were fed normal chow diet (ND) for up to 30 weeks of age. Five- and thirty-week-old WT and CKO mice were individually placed in a PhenoMaster metabolic cage system (TSE Systems, Berlin, Germany) for 3 days. Standard 12 h light-dark cycles were maintained throughout the experiment. Mice were acclimated for 24 h prior experiment. The PhenoMaster system measured O_2_ uptake and CO_2_ production every 12 min for 72 h, and energy expenditure was calculated from these parameters.
2 Seven-week old male C57BL/6J (n = 16) and CKO (n = 16) mice were randomly assigned to four groups (8 mice/group): ND-fed WT and CKO and high-fat diet (HFD)-fed WT and CKO mice. HFD was provided for 4 weeks and contained 60% of calories from fat, 20% from proteins, and 20% from carbohydrates (Research Diet Inc., New Brunswick, NJ, USA).
3 Seven-week-old male CKO mice (n = 30) were fed HFD and randomly divided into three groups (10 mice/group): no treatment, melatonin treatment, and *N*-acetyl cysteine (NAC) treatment. Melatonin (500 μg/kg body weight (B.W.)/day; Sigma-Aldrich, St. Louis, MO, USA) and NAC (60 mg/kg B.W./day; Sigma-Aldrich) were dissolved in saline solution and injected intraperitoneally once a day for 6 weeks.
4 Seven-week-old male CKO mice (n = 24) were fed HFD randomly divided into three groups (8 mice/group): no treatment, GKT137831 treatment, and metformin treatment. GKT137831 (50 mg/kg B.W./day; Biovision, Milipitas, CA, USA) and metformin (100 mg/kg B.W./day; Sigma-Aldrich) were dissolved in saline solution and administered orally once a day for 6 weeks.

In the last week of the experiment, body composition of all mice was measured using the Minispec LF50 equipment (Bruker, Billerica, MA, USA). At the end of the experiment, all mice were anesthetized with isoflurane (5 mg/kg; Hana Pharm., Seoul, Korea) after overnight fasting. Blood was taken from the inferior vena cava to determine fasting plasma free fatty acids, TG, total cholesterol, and glucose concentrations using an enzyme-linked immunosorbent assay kit (Abcam, Cambridge, MA, USA). The epididymal fat was removed, wash out with cold physiological saline, weighed, immediately frozen in liquid nitrogen, and stored at −80 °C until further processing. All animal experiments were carried out in strict accordance with the recommendations of the Guide for the Care and Use of Laboratory Animals of the National Institutes of Health. The protocol was approved by the Keimyung University Institutional Ethics Committee, Daegu, Korea (permit number: KM2016-08 and KM2018-01).

### 2.2 RNA extraction and quantitative polymerase chain reaction (qPCR)

Trizol reagent (Thermo Fisher Scientific, Waltham, MA, USA) was used to extract total RNA from mouse epididymal fat, according to the manufacturer's manual. Total RNA was quantified using a DS-11 spectrophotometer (Denovix, Wilmington, DE, USA). Next, complementary DNA was synthesized using SuperScript® III (Thermo Fisher Scientific) in a reaction containing oligo-dT primers. To quantify gene expression, qPCR was conducted using a StepOnePlus Real-Time PCR system (Applied Biosystems, Foster City, CA, USA) and SYBR Green (Thermo Fisher Scientific). PCR conditions were as follows: pre-denaturation at 95 °C for 5 min; 50 cycles at 94 °C for 10 s, 60 °C for 30 s, and 72 °C for 30 s; and final elongation at 72 °C for 10 min. All samples were measured in duplicate to ensure reproducibility, and gene expression was calculated from the Ct value using the 2^−ΔΔCt^ method [24]. Glyceraldehyde 3-phosphate dehydrogenase (GAPDH) was used as reference gene. Sequences of target gene primers are shown in Table 1.

**Table 1:** Primer sets for qPCR analysis.

Gene	Direction	Primer sequence
GAPDH	Forward Reverse	TGC ACC ACC AAC TGC TTA GC GGC ATG GAC TGT GGT CAT GAG
NOX4	Forward Reverse	GAA GCC CAT TTG AGG AGT CA GGG TCC ACA GCA GAA AAC TC
p22Phox	Forward Reverse	GTC CAC CAT GGA GCG ATG TG CAA TGG CCA AGC AGA CGG TC
CPT1a	Forward Reverse	CCA TCC TGT CCT GAC AAG GTT TAG CCT CAC TTC TGT TAC AGC TAG CAC
CPT1b	Forward Reverse	CGA GGA TTC TCT GGA ACT GC GGT CGC TTC TTC AAG GTC TG
CPT2	Forward Reverse	CAACTCGTATACCCAAACCCAGTC GTTCCCATCTTGATCGAGGACATC
Acox1	Forward Reverse	GCC CAA CTG TGA CTT CCA TT GGC ATG TAA CCC GTA GCA CT
Fgf21	Forward Reverse	CCT CTA GGT TTC TTT GCC AAC CTG GTA CAC ATT GTA ACC GTC
Lcad	Forward Reverse	GTA GCT TAT GAA TGT GTG CAA CTC GTC TTG CGA TCA GCT CTT TCA TTA
Mcad	Forward Reverse	GAT CGC AAT GGG TGC TTT TGA TAG AA AGC TGA TTG GCA ATG TCT CCA GCA AA
Cs	Forward Reverse	GGA GCC AAG AAC TCA TCC TG TCT GGC CTG CTC CTT AGG TA
Aconitase	Forward Reverse	TGG CTG CCA GTA TGA CCA AGT ATG TGG CTT TAG CTC ATT GAG GTT
IDH	Forward Reverse	ATT TTG TGG TAG ATC GAG CTG G CCT CCG GCA GGG AAG TTA TAC
SDH	Forward Reverse	CCT CGA ATG CAG ACG TAC GA CAA CAC CAT AGG TCC GCA CTT
MDH	Forward Reverse	AAG GCT ACC TTG GAC CGG AG CAT CAC AAC CTT TGA GGC AAT CT
Pref-1	Forward Reverse	CTG GCT TCT CAG GCA ACT TC AGG GGT ACA GCT GTT GGT TG
GATA2	Forward Reverse	TGC ATG CAA GAG AAG TCA CC AGA CTG GAG GAA GGG TGG AT
PPARγ	Forward Reverse	CCA GAG TCT GCT GAT CTG CG GCC ACC TCT TTG CTC TGC TC
FAS	Forward Reverse	AGC TTC GGC TGC TGT TGG AAG T TCG GAT GCC TCT GAA CCA CTC ACA
SREBP1c	Forward Reverse	GGA GCC ATG GAT TGC ACA TT GGC CCG GGA AGT CAC TGT
SREBP2	Forward Reverse	CCC TTG ACT TCC TTG CTG CA GCG TGA GTG TGG GCG AAT C

### 2.3 Hematoxylin and eosin (H&E) staining

Epididymal fat was collected from each mouse, fixed in 10% (v/v) paraformaldehyde/phosphate-buffered saline (PBS), embedded in paraffin, and cut into 6 μm-thick sections for staining with H&E. Sections were visualized using an Axio Scope. A1 microscope (Carl Zeiss, Oberkochen, Germany) at 100 × magnification.

### 2.4 Cell culture and differentiation

3T3-L1 cells were kindly provided by Dr. Kim at Yonsei University College of Medicine, Korea. 3T3-L1 cells were cultured Dulbecco's Modified Eagle Medium (DMEM) supplemented with 10% (v/v) calf serum and 1% (v/v) penicillin/streptomycin (Thermo Fisher Scientific) at 37 °C in humidified 5% CO_2_ atmosphere. To induce differentiation, 48 h post-confluent 3T3-L1 cells were provided DMEM supplemented with 10% fetal bovine serum (FBS), 1 μg/mL insulin, 0.5 mM 3-iso-butyl-1-methylxanthine, and 1 μM dexamethasone. Cells were cultured in DMEM supplemented with 10% (v/v) FBS and 1 μg/mL insulin for 2 days. For further cell culture, medium was replaced with DMEM supplemented with 10% (v/v) FBS every 2 days.

Primary mouse embryonic fibroblasts (MEFs) of individual embryos were isolated using MEF isolation kit (Thermo Fisher Scientific), according to the manufacturer's protocol. MEFs were isolated from embryos of C57BL/6J and CKO homozygous mice at 13.5 days post coitum. Embryos were collected and separated from the yolk sac, placenta, head, and red organs. The remaining bodies were washed in Hanks' Balanced Salt Solution without Ca^2+^ and Mg^2+^, dissociated with MEF isolation enzyme (with papain), and incubated at 37 °C for 20 min. After incubation, the MEF isolation enzyme was removed and the complete DMEM for primary cell was added. MEFs were pipetted up and down a few times to make single cells. MEFs were cultured in DMEM supplemented with 10% FBS and 1% penicillin/streptomycin at 37 °C in humidified 5% CO_2_ atmosphere. For adipogenesis induction, 48 h post-confluent MEFs were provided DMEM supplemented with 10% (v/v) FBS, 10 μg/mL insulin, 0.5 mM 3-iso-butyl-1-methylxanthine, and 1 μM dexamethasone. MEFs were cultured in DMEM supplemented with 10% (v/v) FBS and 10 μg/mL insulin for 2 days. Medium was replaced with DMEM supplemented with 10% (v/v) FBS. Experiments were carried out using MEFs at passage two or three.

### 2.5 Treatment and transfection of 3T3-L1 cells

3T3-L1 cells at day 4 of differentiation were transfected with 100 nM control or catalase-small interfering RNA (siRNA; Santa Cruz Biotechnology, Santa Cruz, CA, USA) using Lipofectamine (Thermo Fisher Scientific) in Opti-MEM® medium for 72 h, according to the manufacturer's protocol. Meanwhile, cells were treated with 1 mM metformin and/or 20 μM GKT137831 and used for experiments after 72 h. All experiments were performed in duplicate and repeated at least three times to ensure reproducibility. For early-stage adipogenesis assays, 3T3-L1 cells were transfected with 50 nM control or catalase-siRNA in Opti-MEM® for 24 h at D-2. Then, at D-1, 24 h before mitotic clonal expansion, the medium was replaced with DMEM supplemented with 10% calf serum.

### 2.6 Oil Red O (ORO) staining of MEFs and 3T3-L1 cells

MEFs and 3T3-L1 cells were rinsed with PBS, fixed in 10% formalin at 37 °C and 5% CO_2_, washed with 60% isopropanol, stained with ORO solution (Sigma-Aldrich) for 15 min at 25 °C, and rinsed with PBS to remove unattached ORO. Stained cells were imaged using a DM-IL microscope (Leica, Wetzlar, Germany) at 400 × magnification and extracted using 100% isopropanol for quantification of lipid accumulation. Isopropanol from an empty well was used as control, and the absorbance was measured spectrophotometrically at 510 nm for quantification of ORO staining.

### 2.7 Measurement of H_2_O_2_

ROS were detected using a H_2_O_2_ Assay Kit (Biovision), according to the manufacturer's protocol. All samples were analyzed immediately and/or aliquoted to avoid repeated freeze-thaw cycles. Samples were directly mixed with the assay buffer, OxiRed probe, and horseradish peroxidase solution after filtration using 10 Kd Spin Columns (Biovision). Thereafter, H_2_O_2_ was measured at 570 nm using microplate reader.

### 2.8 Western blot analysis

Protein of epididymal fat was extracted using Tissue Protein Extraction Reagent (T-PER) (Thermo Fisher Scientific) and that of MEFs and 3T3-L1 cells was extracted with the radio-immunoprecipitation assay buffer (Cell Signaling Technology, Danvers, MA, USA), and quantified using the bicinchoninic acid method. Protein extraction from adipose tissue was carried out very carefully. We added 100 μL of T-PER per 0.1 g of adipose tissue, homogenized sufficiently, and then centrifuged at 14,000 *g* for 10 min at 4 °C. The supernatant was taken so that the top layer of fat and bottom pallets do not come with it. This process was repeated at least 2 times to purify it to a clean protein. Total protein (15–45 μg) was subjected to 10% sodium dodecyl sulfate–polyacrylamide gel electrophoresis, transferred to polyvinylidene fluoride membranes (Whatman, Maidstone, UK), blocked, and probed with primary antibodies in 5% skim milk for 24 h at 4 °C. The following primary antibodies (1:1000) were used: rabbit anti-GAPDH, anti-nicotinamide adenine dinucleotide phosphate (NADPH) oxidase 4 (NOX4), anti-p22phox, anti-phospho-adenosine monophosphate (AMP)-activated protein kinase (AMPK) α, anti-AMPKα, anti-nuclear respiratory factor 1 (NRF1), and anti-glucose-6-phosphate dehydrogenase (G6PD) (Cell Signaling Technology); rabbit anti-catalase, anti-mitochondrial transcription factor A (TFAM), and PPARγ coactivator 1-alpha (PGC1α) and mouse anti-total oxidative phosphorylation system (OXPHOS) cocktail containing NADH dehydrogenase [ubiquinone] 1 beta subcomplex subunit 8, mitochondrial (NDUFB8), succinate dehydrogenase (SDH) complex iron sulfur subunit B (SDHB), ubiquinol-cytochrome C reductase core protein 2 (UQCRC), mitochondrially encoded cytochrome C oxidase I (MTCO1), and adenosine triphosphate (ATP) synthase alpha-subunit gene (ATP5A) (Abcam); and rabbit anti-hypoxia-inducible factor 1α (HIF1 α) (Cayman, Ann Arbor, MI, USA). The immunoreactive antigen was then recognized with horseradish peroxidase-labeled anti-rabbit IgG and anti-mouse IgG (Cell Signaling Technology), detected using an ECL kit (Vilber Lourmat, Marne la Vallée, France), and quantified using Image Analyzer (Vilber Lourmat).

### 2.9 Statistical analysis

The parameter values are presented as the mean ± standard error of the mean (SEM), and *n* denotes the number of animals used in each experiment or independent values for *in vitro* experiments. Significant differences were determined via Student's *t*-test using SPSS software (SPSS Inc., Chicago, IL, USA). Results were considered statistically significant at p < 0.05.

## 3 Results

CKO mice are obesity-prone, presenting elevated H_2_O_2_ and NOX4 expression in adipose tissue.

**Fig. 1:**
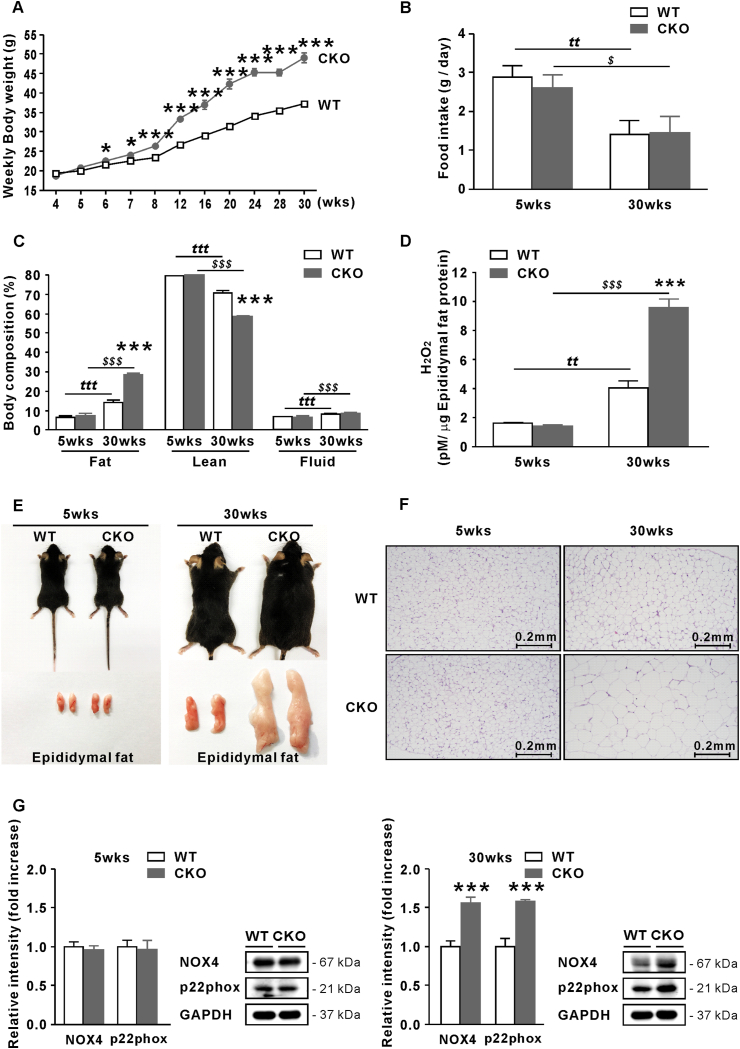
CKO mice are more prone to obesity with age than WT mice. (A) Body weight of WT and CKO mice between 4 and 30 weeks of age. (B) Food intake of WT and CKO mice at 5 and 30 weeks of age. (C) Body composition (%) of WT and CKO mice at 5 and 30 weeks of age. (D) H_2_O_2_ concentration in epididymal fat of WT and CKO mice at 5 and 30 weeks of age. (E) Representative pictures of WT and CKO mice at 5 and 30 weeks of age. (F) H&E staining of epididymal fat of CKO mice at 5 and 30 weeks of age ( × 100). (G) Protein levels of NOX4 and p22phox relative to GAPDH in epididymal fat of WT and CKO mice at 5 and 30 weeks of age. Data are expressed as the mean ± SEM; Student's *t*-test, n = 10, *P < 0.05, **P < 0.01, ***P < 0.001 versus WT mice; ^tt^*P* < 0.01, ^ttt^*P* < 0.001, 5-week-old WT mice versus 30-week-old WT mice; ^$^*P* < 0.05, ^$$$^*P* < 0.001, 5-week-old CKO mice versus 30-week-old CKO mice.

Compared to WT mice, providing ND *ad libitum*, body weight of CKO mice significantly increased from 6 weeks of age (p < 0.05), with an accelerated increase from 8 weeks of age (p < 0.001; Fig. 1A). During breeding up to 30 weeks of age, food intake gradually decreased but did not differ between both groups (Fig. 1B), suggesting the increase in the body weight of CKO mice is not due to increased energy intake but to decreased energy expenditure (Supplementary Fig. 1A). Consistently, 30-week-old CKO mice developed more body fat mass than WT mice (p < 0.001), despite the decrease in lean body mass (p < 0.001; Fig. 1C, E-F), indicating that body weight gain in CKO mice is due to white adipose tissue (WAT) gain. At 30 weeks of age, higher H_2_O_2_ concentrations were observed in the epididymal fat of CKO mice than in that of WT mice (Fig. 1D), but no difference in H_2_O_2_ levels at 5 weeks of age was found between both groups.

NOX4 expression was found to be increased in the WAT of obese mice compared to normal mice, thus facilitating oxidative stress [25]. Additionally, NOX4 expression is stimulated by exogenous H_2_O_2_ in 3T3-L1 cells [25]. Consistently, we found that protein levels of NOX4 and its subunit p22phox were significantly higher in the WAT of 30-week-old CKO mice than in that of age-matched WT mice, whereas these levels were similar in WT and CKO mice at 5 weeks of age (Fig. 1G).

Four weeks of HFD accelerates adiposity in CKO mice, with increased mitochondrial impairment in adipose tissue, which is ameliorated by antioxidants.

**Fig. 2:**
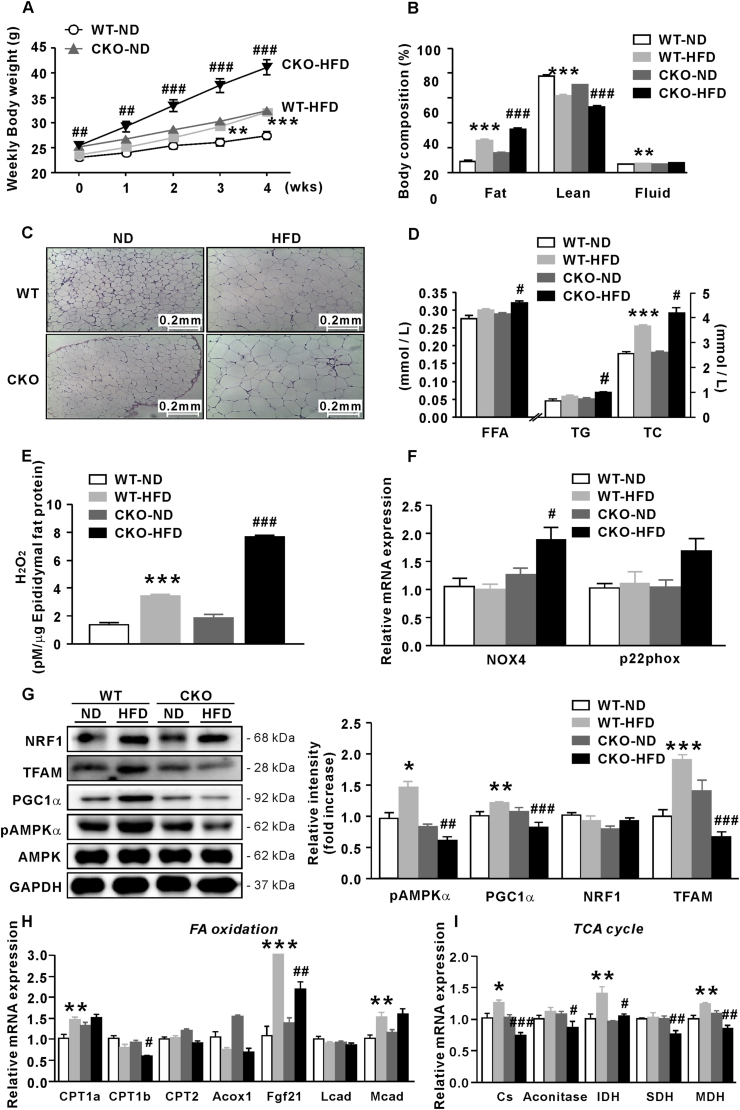
HFD stimulates adipocyte hypertrophy and mitochondrial dysfunction in CKO mice. (A) Body weight of WT and CKO mice fed ND or HFD for 4 weeks. (B) Body composition (%) of WT and CKO mice fed ND or HFD. (C) H&E staining of epididymal fat of WT and CKO mice fed ND or HFD ( × 100). (D) Plasma lipid profiles of WT and CKO mice fed ND or HFD. (E) H_2_O_2_ concentration in epididymal fat of WT and CKO mice fed ND or HFD. (F) Relative mRNA levels of NOX4 and p22phox in epididymal fat of WT and CKO mice fed ND or HFD. (G) Protein levels of PGC1α, TFAM, and NRF1 relative to GAPDH and pAMPKα (relative to AMPKα) in epididymal fat of WT and CKO mice fed ND or HFD. qPCR analysis of (H) FA oxidation- and (I) TCA cycle-related mRNA in epididymal fat of WT and CKO mice fed ND or HFD. Data are expressed as the mean ± SEM; Student's *t*-test, n = 8, *P < 0.05, **P < 0.01, ***P < 0.001 versus ND-fed WT mice; ^#^*P* < 0.05, ^##^*P* < 0.01, ^###^*P* < 0.001 versus HFD-fed WT mice.

To evaluate the effect of excessive H_2_O_2_ on body fat mass, 7-week-old CKO and WT mice were fed HFD for 4 weeks, as HFD induces oxidative stress in WAT [25]. Expectedly, body weight gain was higher in CKO mice than WT mice (Fig. 2A). While HFD-fed WT mice gained more weight than ND-fed WT mice from week 3 (p < 0.01), CKO mice gained significantly more weight than ND-fed CKO mice within a week of HFD administration due to increased body adiposity (Fig. 2B and C). Plasma levels of free fatty acid (FFA) (p < 0.05), TG (p < 0.05), and total cholesterol (p < 0.05) were significantly higher in HFD-fed CKO mice than in HFD-fed WT mice (Fig. 2D). H_2_O_2_ concentration in the epididymal fat of HFD-fed CKO mice was also the highest (p < 0.001) among the four groups (Fig. 2E). Additionally, NOX4 mRNA levels in the epididymal fat of these mice were significantly higher than those of HFD-fed WT mice (Fig. 2F), and the same trend was observed for p22phox mRNA levels (Fig. 2F). Altogether, these results suggest that increased H_2_O_2_ in adipose tissue is positively associated with fat accumulation and NOX4 expression.

To assess whether catalase ablation affects mitochondrial function and biogenesis, proteins associated with mitochondria in epididymal fat were analyzed. PGC1α is a major regulator that induces mitochondrial biogenesis through activating other transcription factors, such as NRF1, which in turn promotes TFAM expression. TFAM is the main transcription factor involved in controlling mitochondrial DNA transcription. AMPKα is a key modulator of mitochondrial biogenesis and a cell energy sensor. Four-week HFD feeding increased the expression of proteins related to mitochondrial biogenesis and function in WT mice (~p < 0.001; Fig. 2G), possibly as an adaptive mechanism. Conversely, mitochondria-associated proteins, including pAMPKα at Thr172, were significantly repressed by HFD in CKO mice compared to WT mice (p < 0.01), suggesting that mitochondria of adipocytes in CKO mice are functionally impaired by excessive H_2_O_2_. To confirm this, mRNA levels of factors involved in FA oxidation (carnitine palmitoyltransferase (CPT)1a, CPT1b, CPT2, acyl-CoA oxidase 1 (Acox1), fibroblast growth factor 21 (Fgf21), long-chain acyl-CoA dehydrogenases (Lcad), and medium-chain acyl-CoA dehydrogenase (Mcad)) (Fig. 2H) and tricarboxylic acid (TCA) cycle (citrate synthase (Cs), aconitase, isocitrate dehydrogenase (IDH), SDH, and malate dehydrogenase (MDH)) (Fig. 2I) were measured in epididymal fat. Consistently, increased fatty acid oxidation- and TCA cycle-related factors were found in WT mice fed HFD for 4 weeks, whereas the opposite was found in HFD-fed CKO mice, corroborating failure of mitochondrial function and biogenesis induced by overwhelming H_2_O_2_ concentration.

**Fig. 3:**
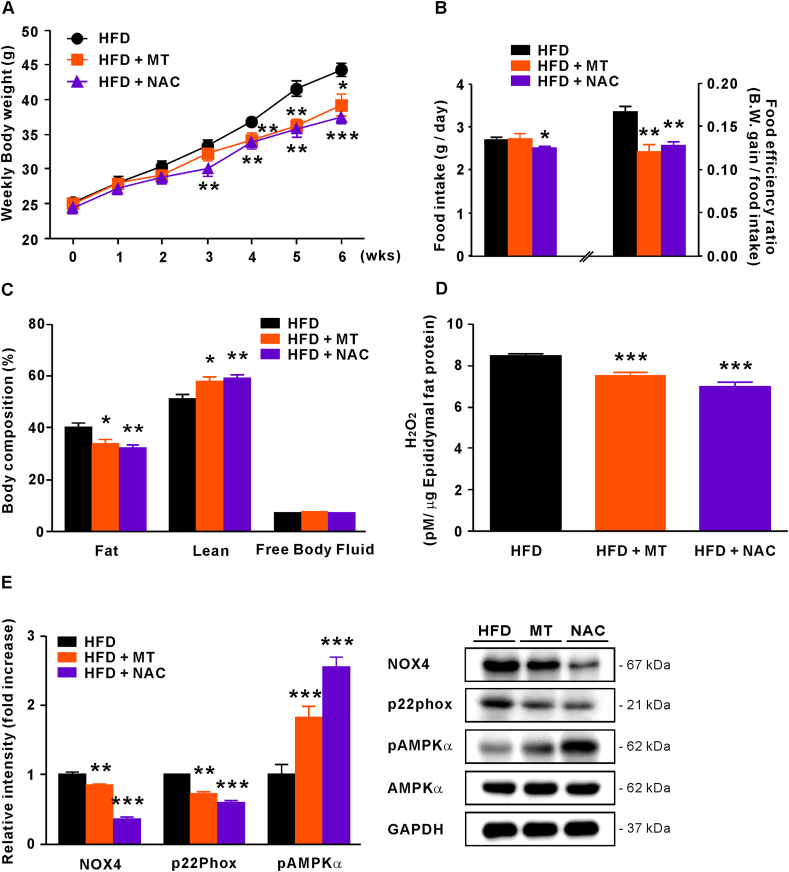
Melatonin and NAC inhibit body weight gain in HFD-fed CKO mice. (A) Body weight of HFD-fed CKO mice treated with melatonin or NAC for 6 weeks. (B) Food intake and FER were measured in each group. (C) Body composition of HFD-fed CKO mice treated with melatonin or NAC. (D) H_2_O_2_ concentration in epididymal fat. (E) Protein levels of NOX4 and p22phox relative to GAPDH and pAMPKα (relative to AMPKα) in epididymal fat. Data were expressed as the mean ± SEM; Student's *t*-test, n = 10, *P < 0.05, **P < 0.01, ***P < 0.001 versus HFD-fed CKO mice.

To determine if obesity is indeed accelerated by ROS overload, we administered the antioxidants melatonin (500 μg/kg B.W./day) or NAC (60 mg/kg B.W./day) to HFD-fed CKO mice for 6 weeks. Body weight increase in CKO mice was significantly ameliorated by these antioxidants from week 3 of administration (Fig. 3A). NAC-treated CKO mice appeared to present lower dietary intake, but their food efficiency ratio (FER) was significantly lower than that of untreated CKO mice, as in the melatonin-treated group (Fig. 3B), suggesting that energy expenditure is ameliorated by antioxidant treatment. Consistently, NAC- and melatonin-treated CKO mice presented significantly decreased fat mass (p < 0.05 and p < 0.01, respectively) and increased lean mass (p < 0.05 and p < 0.01, respectively; Fig. 3C). We also analyzed H_2_O_2_ concentration in epididymal fat from each mouse group. Expectedly, the melatonin- and NAC-treated groups exhibited significantly lower H_2_O_2_ concentrations than the untreated group (p < 0.001; Fig. 3D). Furthermore, NOX4 and p22phox protein levels in adipose tissue were decreased after melatonin (p < 0.01) and NAC (p < 0.001) treatment, and these antioxidants significantly increased phosphorylated AMPKα (p < 0.001; Fig. 3E). Along with the previous results, this suggests that excessive H_2_O_2_ is critical for the development of obesity and mitochondrial impairment.

Catalase knockdown promotes adipogenesis in 3T3-L1 cells and MEFs through elevated H_2_O_2_

**Fig. 4:**
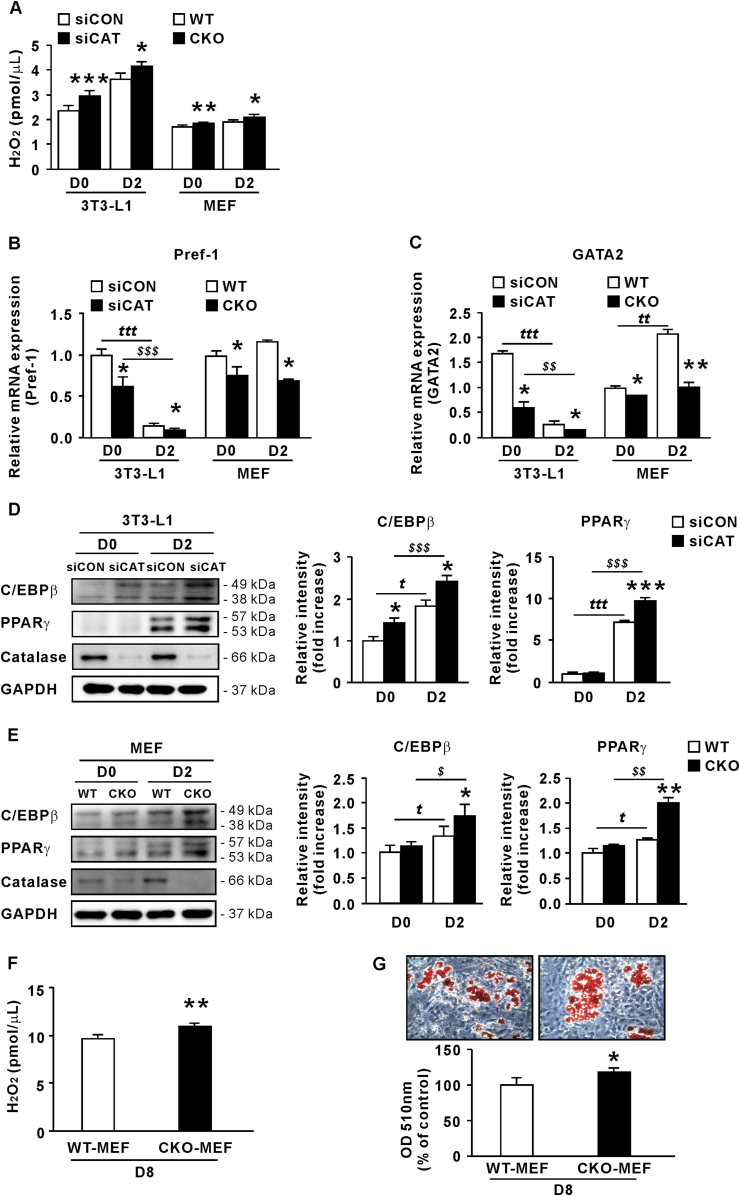
Catalase knockdown in 3T3-L1 cells and MEFs promotes adipogenesis. (A) H_2_O_2_ concentration during early-stage differentiation of 3T3-L1 cells transfected with siCAT and CKO MEFs (n = 6). (B) Pref-1 and (C) GATA2 mRNA levels (n = 3). (D) Protein levels of catalase, C/EBPβ, and PPARγ relative to GAPDH in siCAT-transfected 3T3-L1 cells (n = 4). (E) Protein levels of catalase, C/EBPβ, and PPARγ relative to GAPDH in CKO MEFs (n = 4). (F) H_2_O_2_ concentration in MEFs from WT and CKO mice at D8 (n = 3). (G) ORO staining of MEFs from WT and CKO mice at D8 (n = 3). Data are expressed as the mean ± SEM; Student's t-test, *P < 0.05, **P < 0.01, ***P < 0.001 versus siCON-transfected 3T3-L1 cells or WT MEFs; ^t^*P* < 0.05, ^tt^*P* < 0.01, ^ttt^*P* < 0.001, siCON-transfected 3T3-L1 cells or WT MEFs at D0 versus siCON-transfected 3T3-L1 cells or WT MEFs at D2; ^$^*P* < 0.05, ^$$^*P* < 0.01, ^$$$^*P* < 0.001, siCON-transfected 3T3-L1 cells or WT MEFs at D0 versus siCAT-transfected 3T3-L1 cells or CKO MEFs at D2.

To determine whether catalase knockdown affects adipocyte differentiation, 3T3-L1 cells were transfected with siRNA prior to differentiation (for 24 h at D-2). Additionally, we analyzed MEFs derived from WT and CKO embryos. In catalase-deficient 3T3-L1 cells and MEFs, H_2_O_2_ production was significantly higher at D0 and D2 than in each control group (Fig. 4A). mRNA levels of preadipocyte factor 1 (Pref-1) and GATA-binding protein 2 (GATA2), important preadipocyte markers [26,27], were also significantly reduced at D0 and D2 in catalase-siRNA (siCAT)-transfected 3T3-L1 cells and CKO MEFs compared to each control group (Fig. 4B and C). Furthermore, Pref-1 and GATA2 mRNA levels in 3T3-L1 cells gradually decreased at D2 compared to D0, which was regarded as loss of preadipocyte characteristics, and thus differentiation into adipocytes. However, these MEFs were different and, unlike the 3T3-L1 cells, which exclusively present hallmarks of preadipocytes, presented heterogeneous properties, being able to differentiate into osteoblasts, chondrocytes, and adipocytes. Thus, the used adipocyte inducer may trigger MEFs to differentiate to preadipocyte lineages in the first place [28]. Therefore, Pref-1 and GATA2 mRNA levels in WT MEFs might be temporarily increased at 2 days of differentiation compared to those at D0.

Moreover, C/EBPβ and PPARγ are critical for differentiation into mature adipocytes [29,30], and we confirmed that the levels of these transcription factors increased over time in control and catalase-deficient 3T3-L1 cells and MEFs (Fig. 4D and E, respectively), indicating that differentiation was successfully induced. Expectedly, C/EBPβ and PPARγ protein levels in catalase-deficient cells were significantly higher than those in control cells, particularly at D2. Compared to WT MEFs, H_2_O_2_ levels at D8 were significantly elevated in CKO MEFs (p < 0.01; Fig. 4F). The resulting increased adipogenesis was observed in CKO MEFs at D8 using ORO staining (p < 0.05; Fig. 4G). These results suggest that the increase in H_2_O_2_ induced by catalase knockdown promotes cell differentiation toward mature adipocytes.

### 3.1 Elevated H_2_O_2_ induces adipocyte hypertrophy with NOX4 and AMPKα regulation

**Fig. 5:**
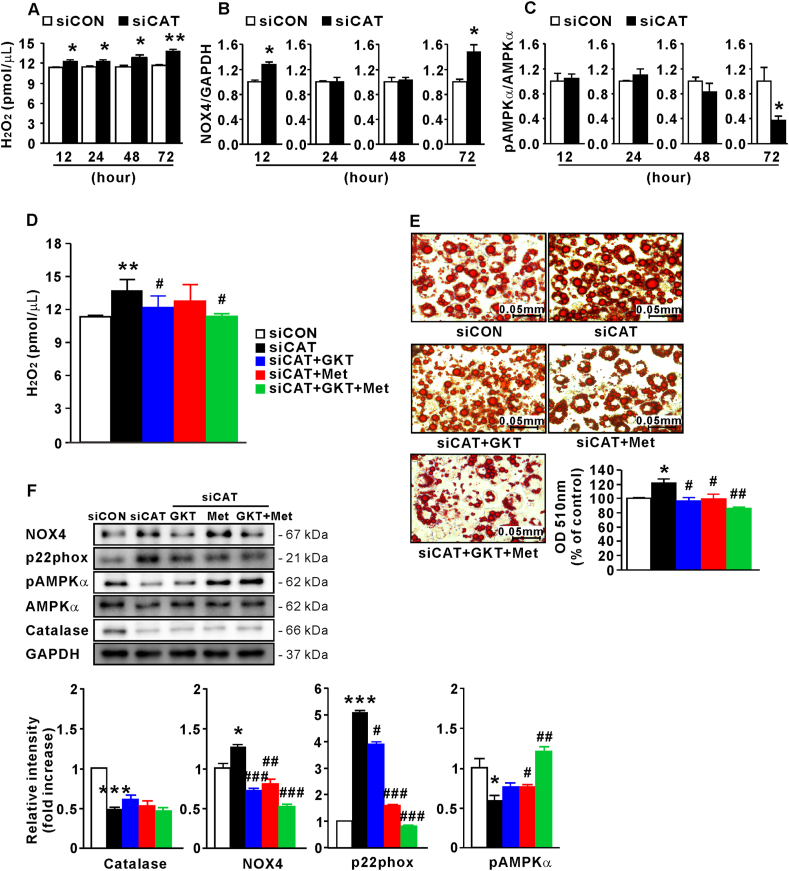
H_2_O_2_ increases adipocyte hypertrophy with NOX4 and AMPKα modulation. (A) H_2_O_2_ concentration in 3T3-L1 cells according to siCAT transfection time (n = 4). (B) Protein levels of NOX4 relative to GAPDH and (C) pAMPKα (relative to AMPKα) in 3T3-L1 cells according to siCAT transfection time (n = 3–6). (D) H_2_O_2_ concentration in siCAT-transfected 3T3-L1 cells treated with GKT137831 or/and metformin (n = 4). (E) ORO staining of siCAT-transfected 3T3-L1 cells treated with GKT137831 or/and metformin (n = 3). (F) Protein levels of catalase, NOX4, and p22phox relative to GAPDH and pAMPKα (relative to AMPKα) in siCAT-transfected 3T3-L1 cells treated with GKT137831 or/and metformin (n = 3–6). Data are expressed as the mean ± SEM; Student's *t*-test, *P < 0.05, **P < 0.01, ***P < 0.001 versus siCON-transfected 3T3-L1 cells; #P < 0.05, ##P < 0.01, ###P < 0.001 versus siCAT-transfected 3T3-L1 cells.

To confirm whether excessive H_2_O_2_, resulting from catalase deficiency, also induces adipocyte hypertrophy, 3T3-L1 cells were transfected with siCAT at D4 for 2 days, and the degree of lipogenesis, H_2_O_2_ production, and protein levels of NOX4 and AMPKα were analyzed at D7. H_2_O_2_ levels increased by siCAT were detected after 12 h (p < 0.05; Fig. 5A) and maintained throughout the experiment. NOX4 protein levels (Western blot bands not shown) were elevated at 12 and 72 h (p < 0.05), but no difference was found in these levels at 24 and 48 h relative to control-siRNA (siCON)-transfected cells (Fig. 5B). AMPKα phosphorylation (Western blot bands not shown) was not significantly different between both cell groups until 48 h, decreasing 72 h after siCAT transfection (p < 0.05; Fig. 5C). These results suggest that the increase in H_2_O_2_ and H_2_O_2_-induced NOX4 expression might occur earlier than the decrease in pAMPKα, indicating that excessive ROS probably inhibit AMPKα activity, which supports the mitochondrial function and biogenesis [31].

Based on these results, we treated siCAT-transfected 3T3-L1 cells with GKT137831 (20 μM), a NOX4 inhibitor, and metformin (1 mM), an AMPKα activator, for 72 h from D4. At D7, H2O2 concentration, which was increased by siCAT, was reduced by GKT137831 (p < 0.05; Fig. 5D). Although no difference in H_2_O_2_ concentration was found when metformin was administered alone to siCAT-transfected 3T3-L1 cells, a significant decrease was observed when GKT137831 and metformin were administered together (p < 0.01). Moreover, lipid accumulation was higher in siCAT-transfected 3T3-L1 cells than in control siRNA-transfected cells (p < 0.05; Fig. 5E). Treatment of siCAT-transfected cells with GKT137831 or metformin reduced lipid accumulation (p < 0.05), which was further reduced upon treatment with both substances (p < 0.01). At D7, we confirmed that catalase knockdown increased NOX4 (p < 0.05) and p22phox (p < 0.001) protein levels and reduced AMPKα phosphorylation (p < 0.05; Fig. 5F), indicating that excessive H_2_O_2_ facilitates adipocyte hypertrophy and modulates NOX4 and AMPK signaling. Additionally, these results show that NOX4 inhibition tends to increase AMPK activity (though not significantly), and AMPK activation inhibits NOX4 activity (Fig. 5F), as previously suggested [32].

### 3.2 GKT137831 and metformin ameliorate propensity for obesity in HFD-fed CKO mice

**Fig. 6:**
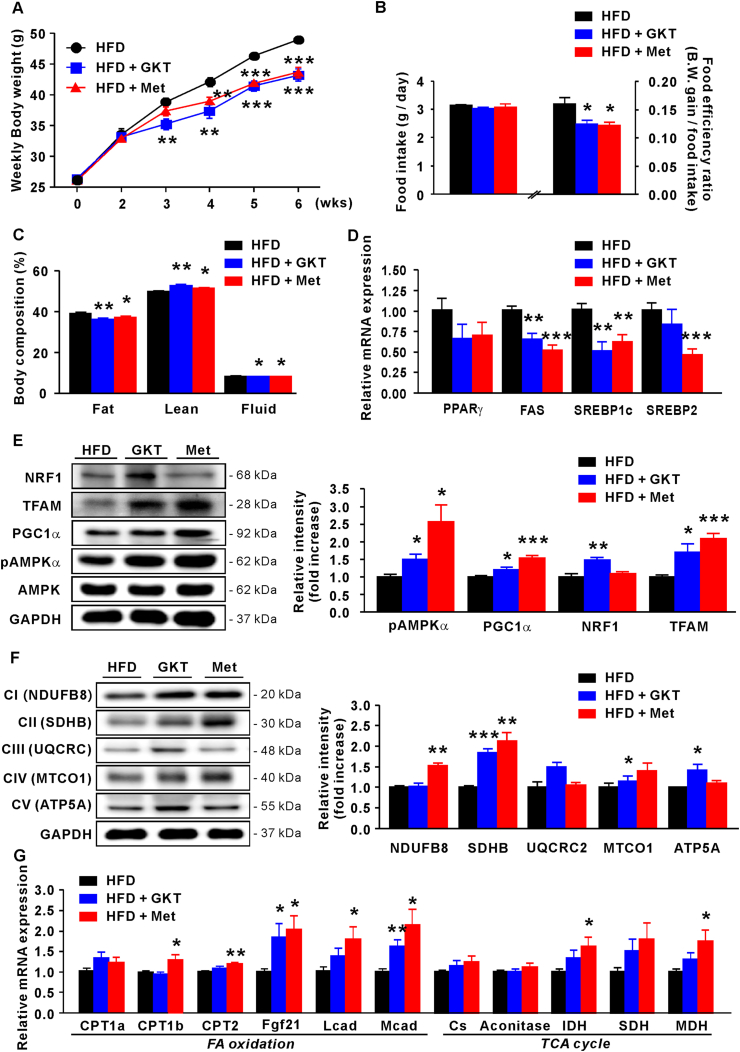
GKT137831 and metformin rescue the propensity for obesity of HFD-fed CKO mice. (A) Body weight of HFD-fed CKO mice treated with GKT137831 or metformin for 6 weeks. (B) Food intake and FER in each group. (C) Body composition of HFD-fed CKO mice treated with GKT137831 or metformin. (D) qPCR analysis of lipogenesis-related mRNA in epididymal fat. (E) Protein levels of PGC1α, TFAM, and NRF1 relative to GAPDH and pAMPKα (relative to AMPKα) in epididymal fat. (F) Western blot analysis of OXPHOS-associated signals, including NDUFB8, SDHB, UQCRC, MTCO1, and ATP5A, in eipididymal fat. (G) qPCR analysis of FA oxidation- and TCA cycle-related mRNA in epididymal fat. Data are expressed as the mean ± SEM; Student's *t*-test, n = 8, *P < 0.05, **P < 0.01, ***P < 0.001 versus HFD-fed CKO mice.

We administered GKT137831 (50 mg/kg B.W./day) or metformin (100 mg/kg B.W./day) to HFD-fed CKO mice to validate the *in vitro* results (Fig. 5). These drugs significantly decreased body weight (p < 0.001), FER (p < 0.05), and fat weight (p < 0.01 and 0.05) and increased lean mass (p < 0.01 and 0.05) after 6 weeks (Fig. 6A–C). Furthermore, we found decreased lipogenic gene expression in the epididymal fat of GKT137831-and metformin-treated HFD-fed CKO mice (Fig. 6D). The expression of sterol regulatory element-binding protein (SREBP)1c (p < 0.01) and its target gene, fatty acid synthase (FAS), was significantly reduced in HFD-fed CKO mice treated with GKT137831 or metformin (p < 0.01 and 0.001, respectively), and that of SREBP2 was dramatically decreased by metformin (p < 0.001; Fig. 6D).

Next, we evaluated the expression of mitochondria-related genes and proteins in HFD-fed CKO mice upon treatment with GKT137831 and metformin, observing a significant amelioration of pAMPKα (p < 0.05 and 0.05, respectively), PGC1α (p < 0.05 and 0.001, respectively), NRF1 (p < 0.01 with GKT137831), and TFAM (p < 0.05 and 0.001, respectively) protein levels in these mice (Fig. 6E). We further assessed the relative abundance of several OXPHOS protein subunits, which showed a trend to increase upon treatment with GKT137831 and metformin (Fig. 6F). Notably, all complex chains except CIII were significantly elevated in the GKT137831-treated group, and metformin markedly increased CI and CII. Furthermore, we measured the mRNA levels of metabolic genes involved in FA oxidation (CPT1a, CPT1b, CPT2, Acox1, Fgf21, Lcad and Mcad) and TCA cycle (Cs, IDH, SDH, and MDH), as markers of mitochondrial function. Fgf21 (p < 0.05) and Mcad (p < 0.01) mRNA levels were significantly higher in the GKT137831-treated group than in the untreated group, and metformin treatment dramatically increased those of CPT1b (p < 0.05), CPT2 (p < 0.01), Fgf21 (p < 0.05), Lcad (p < 0.05), and Mcad (p < 0.05) (Fig. 6G). Moreover, metformin promoted IDH (p < 0.05) and MDH (p < 0.05) expression. These data indicate that catalase activity in WAT profoundly impacts normal mitochondrial function by adjusting the oxidative stress capacity.

CKO mice present reduced overall energy consumption and adipocytes with elevated G6PD and HIF1α expression.

As the CKO mice used in this study was not conditional, we evaluated the basal energy consumption of 30-week-old WT and CKO mice. Expectedly, energy expenditure, CO_2_, and O_2_ consumption were lower in ND-fed CKO mice than in ND-fed WT mice (Supplementary Fig. 1A), suggesting that tissues or organs of CKO mice use significantly less energy than those of WT mice, which can transfer and store energy in WAT. Consistently, fasting plasma glucose levels were higher in CKO mice than in WT mice (Supplementary Fig. 1B). G6PD is the rate-limiting enzyme of the pentose phosphate pathway (PPP) producing NADPH, which is a major pathway in WAT to store TG via fatty acid synthesis [[33], [34], [35]]. G6PD activation occurs when the energy surplus in adipocytes is present. Expectedly, G6PD expression was higher in CKO mice than in WT mice (Supplementary Fig. 1C). Adipocyte hypertrophy can induce hypoxia in adipose tissue and thus HIF1α expression, which supports NOX4 activity and hinders gene transcription associated with mitochondrial biogenesis and function [36], possibly interfering with lipid catabolism. It is widely suggested that adipose tissues are poorly oxygenated in obese humans and mice, resulting in HIF1α induction [37]. Hence, we evaluated HIF1α protein levels and found that they were higher in the adipose tissue of CKO mice than in that of WT mice (Supplementary Fig. 1D). Altogether, these results suggest that G6PD and HIF1α activation further affects ROS-mediated NOX4 activation and mitochondrial dysfunction and potentiates lipogenesis in adipocytes.

## 4 Discussion

CKO mice are susceptible to obesity and obesity-related metabolic diseases [19,[38], [39], [40]]. Consistent with the results of Heit et al. [19], we found that the body weight of CKO mice significantly increased with age compared to WT mice due to their increased fat mass content and hypertrophic adipocytes.

Generally, after treatment with differentiation inducers for 2 days, fat droplets can be observed from D3, and adipocytes are considered fully differentiated from D8. In this process, the differentiation from preadipocytes to adipocytes is called adipogenesis, and the formation and accumulation of lipid droplets after differentiation is called lipogenesis. C/EBPβ and PPARγ are important transcription factors in the process of adipogenesis and lipogenesis [4,29]. In Kim et al.‘s report, treatment with exogenous H_2_O_2_ (100 μM) caused dual phosphorylation of C/EBPβ, which promotes adipogenesis, and H_2_O_2_ treatment (100 μM) also increases fat accumulation [8]. In fat-specific PPARγ-ΚΟ mice, fat droplet formation is suppressed, WAT is hardly observed even at 3 months of age, and embryonic stem cells without the PPARγ gene do not differentiate into adipocytes [30]. Conversely, PPARγ overexpression increases fat accumulation in NIH-3T3 cells [41]. Furthermore, GATA2 and Pref-1 are important preadipocyte markers, and GATA2 overexpression in 3T3-L1 cells inhibits adipocyte differentiation [26,27]. We found that catalase deficiency increased C/EBPβ and PPARγ protein levels and decreased GATA and Pref-1 mRNA levels, promoting adipogenesis and lipogenesis in MEFs and 3T3-L1 cells. Lipogenesis was shown to be increased and decreased in 3T3-L1 cells by H_2_O_2_ (50 μM) and NAC (10 mM), a ROS scavenger, treatment, respectively [9]. These results further support our argument that increased H_2_O_2_ due to catalase deficiency not only promotes adipogenesis but also increases lipogenesis.

ROS generation in adipocytes occurs naturally, but more severely in obese animals [25]. Melatonin and NAC are antioxidants that reduce oxidative stress [[42], [43], [44]] and remove H_2_O_2_, a target of catalase among various ROS [[45], [46], [47]]. Here, administration of these antioxidants inhibited body weight gain and reversed the body composition worsened by HFD in CKO mice. Additionally, H_2_O_2_ concentration in epididymal fat, which was increased 2-fold by HFD compared to WT mice, was decreased by melatonin or NAC administration. Taken together, it suggests that high H_2_O_2_ concentrations may exacerbate the gain of adiposity and thus obesity.

The NOX family encloses proteins that transfer electrons across biological membranes and their primary biological function is ROS production [48], with both NOX4 and NOX2 isoforms being found in adipose tissue [25,49]. In rat adipocytes, H_2_O_2_ production decreases when NOX4 expression is artificially lowered [50]. Additionally, NOX4 mRNA levels increase in 3T3-L1 cells in a H_2_O_2_ concentration-dependent manner [25]. H_2_O_2_ generation by NOX4 is related to PPP. Glycolytic intermediates are used as energy sources or substrates of G6PD, the rate-limiting enzyme of PPP, for NADPH and fatty acid synthesis [33,34]. The superoxide anion is generated when NADPH is oxidized to NADP by NOX4, and then superoxide dismutase promotes the dismutation of superoxide to produce H_2_O_2_ [51,52]. Adipocytes of CKO mice cannot rapidly remove H_2_O_2_, which may increase intracellular oxidative stress. Accordingly, G6PD protein levels were not significantly different between WT and CKO mice at 5 weeks of age, but significantly increased in the epididymal fat of older CKO mice. This is consistent with the increased plasma glucose and free fatty acids in catalase-deficient mice, with or without HFD, which is probably due to low overall energy consumption. Energy surplus in adipocytes can also occur due to ROS-mediated mitochondrial damage. Taken together, it is plausible that oxidative stress in catalase-deficient adipocytes is additive owing to NOX4 activation by excessive ROS itself and increased PPP activity. As mentioned, the increased activity of PPP generates more fatty acids, the substrates of lipogenesis in adipocytes.

HIF1α induces glycolysis-related gene expression to produce energy when oxidative phosphorylation in mitochondria is insufficient [53]. HIF1α is a protein expressed in response to hypoxia and is closely related to obesity [54]. Diebold et al. confirmed that HIF1α binds to the NOX4 gene and that NOX4 induction by HIF1α contributes to maintaining ROS levels in pulmonary artery smooth muscle cells [55]. Here, HIF1α protein levels in epididymal fat did not differ among mouse strains at 5 weeks of age, but in older CKO mice, its levels increased more than 2-fold compared to WT mice. These findings show that HIF1α also contributed to the increase in NOX4 activity in this study. In this context, several studies have reported that NOX4 and AMPKα are inversely correlated in cardiac ventricular tissue, renal mesangial cells and podocytes [32,56,57]. Our work also shows that NOX4 hyperactivation leads to disturbances in AMPKα phosphorylation in adipocytes. The basis of this speculation is the increased NOX4 expression occurring 12 h after siCAT transfection, whereas the decreased AMPKα phosphorylation occurs after 72 h. This supports the negative relationship between NOX4 and AMPKα demonstrated by Eid et al. [32]. Thus, the H_2_O_2_ increase caused by catalase deficiency and NOX4 activation is thought to degrade AMPKα activity, which impairs mitochondrial biogenesis and function, since no findings have proven the direct interaction between NOX4 and AMPKα. The present study demonstrated the pharmacological efficacy of GKT137831 or metformin in reducing NOX4 activity and increasing AMPKα activity to improve the metabolic function of adipocytes.

Pérez-Estrada et al. recently reported that the decreased lifespan of catalase-lacking mice is related to lipid metabolism rather than oxidative damage [58]. Regardless of the diet, weight gain in *Cat*^−/-^ mice is similar to that of WT mice, and fat accumulation is even reduced compared to WT mice. As these authors mentioned, the opposite phenomenon to that previously reported in CKO mice [19,38,39] is probably explained by the different used strains. While most studies have so far used CKO mice with C57BL/6J background, Pérez-Estrada et al. used a C57BL/6 N strain. The biggest difference between these two strains is the insufficient nicotinamide nucleotide transhydrogenase in C57BL/6J mice, which prevents adequate metabolization of peroxide, resulting in redox abnormalities in mitochondria [59]. In other words, even with comparable metabolism, ROS production depends on the strain, and in the case of catalase-mutated C57BL/6 N mice, H_2_O_2_ levels in the body are relatively low, which leads us to believe that ROS-induced damage is not significant.

## 5 Conclusions

We speculate that H_2_O_2_ accumulation due to catalase deletion in WAT induces both adipogenesis and lipogenesis. Catalase deficiency-driven oxidative stress appears to increase differentiation of preadipocytes into adipocytes. For lipogenesis, increased oxidative stress in WAT via increased H_2_O_2_ leads to impaired mitochondrial biogenesis and function, and thus increased lipid synthesis rather than lipid oxidation. Furthermore, energy surplus into adipocytes of CKO mice activates G6PD for lipid synthesis. Thus, H_2_O_2_-mediated oxidative stress may be an instigator and an important cause of obesity. The control of the H_2_O_2_ redox balance and/or NOX4 and AMPKα activity in white adipocytes may be a useful target for new therapies for obesity, as demonstrated in this *in vivo* and *in vitro* study.

## Sources of funding

This study was supported by grants from the 10.13039/501100003725National Research Foundation of Korea (10.13039/501100003725NRF) Grant funded by the Korean Government (10.13039/501100003621MSIP) (No. 2018R1D1A1B07043068 and 2018R1A2B2004429) and the Korea Health Technology R&D Project through the 10.13039/501100003710Korea Health Industry Development Institute (10.13039/501100003710KHIDI) funded by the 10.13039/501100003625Ministry of Health and Welfare, Republic of Korea (No. HI14C1324).

## Author contributions

Shin SK and Cho HW performed most of experiments, analyzed data, and wrote the paper. Song SE performed some experiments, Im SS analyzed data. Bae JH reviewed the draft and revised manuscript. Song DK designed study, analyzed data, and wrote the paper.

## Declaration of competing interest

The authors declare that they have no conflict of interest.

## Appendix A Supplementary data

Multimedia component 1 Multimedia component 1

figs1 figs1
