# Supplementary material for: Oxidative stress resulting from the removal of endogenous catalase induces obesity by promoting hyperplasia and hypertrophy of white adipocytes
Source: Redox Biol. 2020 Oct 10;37:101749. doi: 10.1016/j.redox.2020.101749 (PMC7575809; doi:10.1016/j.redox.2020.101749)
Supplement: Multimedia component 1 [file mmc1.docx]

**Legend of supplementary figure**

**Supplementary Fig. 1.** (A) O_2_ consumption (VO_2_), CO_2_ production (VCO_2_), respiratory exchange ratio (RER), and energy expenditure (EE) of WT and CKO mice at 5 and 30 weeks of age. (B) Plasma glucose and protein levels of (C) G6PD and (D) HIF1α relative to GAPDH in epididymal fat of WT and CKO mice at 5 and 40 weeks of age. Data are expressed as the mean ± SEM; Student’s *t*-test, n = 6, ^*^*P* < 0.05, ^**^*P* < 0.01, ^***^*P* < 0.001 versus WT mice; ^ttt^*P* < 0.001, 5-week-old WT mice versus 40-week-old WT mice; ^$$$^*P* < 0.001, 5-week-old CKO mice versus 40-week-old CKO mice.
