# Supplementary figures and images for: Oxidative stress resulting from the removal of endogenous catalase induces obesity by promoting hyperplasia and hypertrophy of white adipocytes
Source: Redox Biol. 2020 Oct 10;37:101749. doi: 10.1016/j.redox.2020.101749 (PMC7575809; doi:10.1016/j.redox.2020.101749)

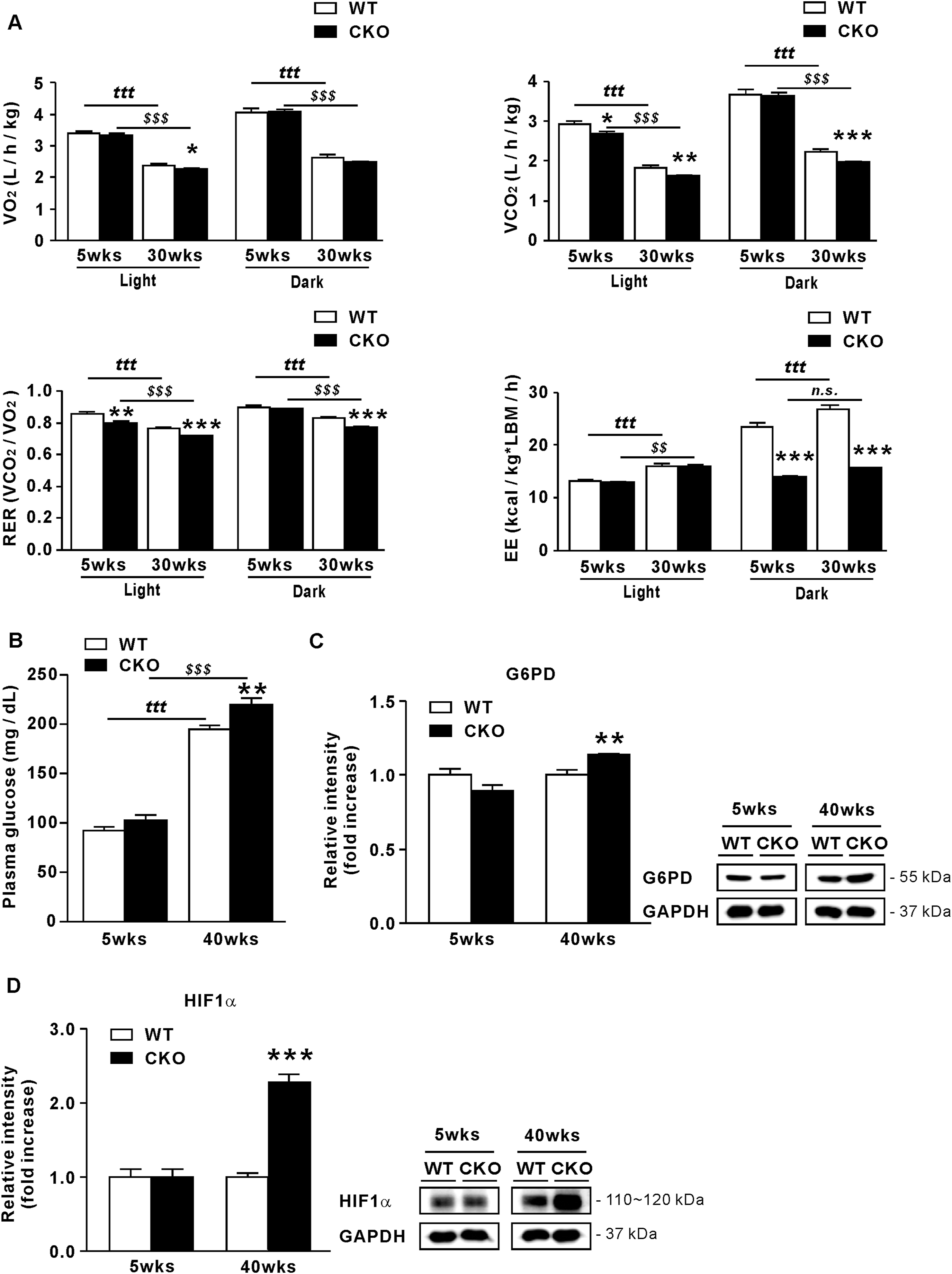

Supplement: figs1 [file mmcfigs1.jpg]
